# miR-146a impedes the anti-aging effect of AMPK via NAMPT suppression and NAD^+^/SIRT inactivation

**DOI:** 10.1038/s41392-022-00886-3

**Published:** 2022-03-04

**Authors:** Hui Gong, Honghan Chen, Peng Xiao, Ning Huang, Xiaojuan Han, Jian Zhang, Yu Yang, Tiepeng Li, Tingting Zhao, Haoran Tai, Weitong Xu, Gongchang Zhang, Chuhui Gong, Ming Yang, Xiaoqiang Tang, Hengyi Xiao

**Affiliations:** 1 The Lab of Aging Research, National Clinical Research Center for Geriatrics, State Key Laboratory of Biotherapy, West China Hospital, Sichuan University, 1 Keyuan 4 Road, Gaopeng Avenue, Chengdu, 610093 China; 2 Key Laboratory of Birth Defects and Related Diseases of Women and Children of MOE, State Key Laboratory of Biotherapy, West China Second University Hospital, Sichuan University, Chengdu, 610041 China

**Keywords:** Senescence, Ageing

## Abstract

Nicotinamide adenine dinucleotide (NAD^+^) is indispensable for the anti-aging activity of the sirtuin (SIRT) family enzymes. AMP-activated protein kinase (AMPK) upregulates NAD^+^ synthesis and SIRT activity in a nicotinamide phosphoribosyltransferase (NAMPT)-dependent manner. However, the molecular mechanisms that affect AMPK-driven NAMPT expression and NAD^+^/SIRT activation remain unclear. In this study, we tried to identify senescence-associated microRNAs (miRNAs) that negatively regulate the cascade linking AMPK and NAMPT expression. miRNA-screening experiments showed that the expression of miR-146a increased in senescent cells but decreased following AMPK activation. Additionally, miR-146a overexpression weakened the metformin-mediated upregulation of NAMPT expression, NAD^+^ synthesis, SIRT activity, and senescence protection, whereas treatment with the miR-146a inhibitor reversed this effect. Importantly, these findings were observed both in vitro and in vivo. Mechanistically, miR-146a directly targeted the 3′-UTR of *Nampt* mRNA to reduce the expression of NAMPT. AMPK activators metformin and 5-aminoimidazole-4-carboxamide (AICAR) hindered miR-146a expression at the transcriptional level by promoting IκB kinase (IKK) phosphorylation to attenuate nuclear factor-kappaB (NF-κB) activity. These findings identified a novel cascade that negatively regulates the NAD^+^/SIRT pathway by suppressing miR-146a-mediated NAMPT downregulation. Furthermore, our results showed that miR-146a impedes the anti-aging effect of AMPK. This mutual inhibitory relationship between miR-146a and AMPK enriches our understanding of the molecular connections between AMPK and SIRT and provides new insight into miRNA-mediated NAD^+^/SIRT regulation and an intervention point for the prevention of aging and age-related diseases.

## Introduction

Aging is characterized by an irreversible decline in biological function, adaptability, and resistance. Mechanistic studies of aging can provide important theoretical support for resisting or delaying the aging process. To date, several aging theories have been postulated, including the oxidative stress/mitochondrial damage theory, telomerase theory, autophagy-inactivation theory, stem cell-depletion theory, and metabolic imbalance theory.^1–3^ Among these, the study of the metabolic imbalance theory has developed rapidly, with evidence showing that caloric restriction can delay aging and prolong the lifespan of many animals.^4,5^ Based on this theory, relevant molecules and signaling pathways have been discovered. Among them, the AMP-activated protein kinase (AMPK) pathway and nicotinamide adenine dinucleotide (NAD^+^)-dependent Sirtuin (SIRT) pathway significantly affect the occurrence and development of aging.^4–6^ However, the relationship between AMPK and SIRT has not been fully elucidated.

The SIRT family has seven members and represents a type of histone deacetylase that uses NAD^+^ as a co-enzyme.^7,8^ SIRTs regulate cellular senescence, proliferation, differentiation, energy metabolism, and other functions.^7,9^ Specifically, SIRT1 inhibits the occurrence of aging and related diseases.^10^ Studies also found that the activity of SIRT1 and the level of cellular NAD^+^ decreased during the aging process, and the supplementation of NAD^+^ precursors can restore NAD^+^ levels and inhibit the occurrence and development of aging^11,12^ or age-related diseases.^13–15^ Additionally, AMPK exerts an anti-aging effect by activating NAD^+^/SIRT1;^16,17^ however, the associated mechanism has not been fully elucidated. One hypothesis for the AMPK-regulated NAD^+^ synthesis is based on the observation that AMPK can upregulate the levels of NAD^+^ by increasing the expression of nicotinamide phosphoribosyltransferase (NAMPT).^6,16^

NAMPT, a rate-limiting enzyme of NAD^+^ synthesis in the salvage pathway, catalyzes the conversion of nicotinamide to nicotinamide mononucleotide, thereby playing a significant role in regulating intracellular NAD^+^ level.^6^ Previous studies showed that NAMPT activity and expression are reduced in senescent tissues and cells,^16,18^ whereas NAMPT overexpression increases cell proliferation and extends the replicative lifespan,^19^ indicating the importance of regulating NAMPT expression during aging. Regarding the regulation of NAMPT expression, previous studies have mostly focused on the transcriptional level, and the results indicate that NAMPT expression can be regulated by transcription factors such as forkhead box O1 (FOXO1), CLOCK, and MYC.^18,20,21^ However, none of these studies were performed under conditions of aging and related cellular processes. Additionally, recent studies show that the mRNA levels of *Nampt* can be regulated by microRNAs (miRNAs) in various physiological and pathological processes.^22,23^ These facts prompted us to hypothesize that miRNAs may be implicated in AMPK-driven NAMPT expression and NAD^+^/SIRT activation, and these miRNAs may potentially be involved in aging and related diseases.

Previous studies offer insight into the specific relationship between AMPK and miRNA.^24,25^ AMPK activator 5-aminoimidazole-4-carboxamide-1-β-D- ribofuranoside (AICAR) changes the miRNA-expression profile in the mouse liver^24^ and another AMPK activator metformin increases the expression of Dicer1, a key miRNA-processing enzyme, in senescent cells.^25^ However, the regulatory mechanisms underlying the relationship between AMPK and aging-related miRNAs are not fully understood.

In the present study, we performed miRNA screening to explore the miRNA-associated mechanism that affects AMPK-mediated aging protection. The study started by screening differentially expressed miRNAs, and particularly explored the implication of miR-146a in NAMPT expression and the suppressive role of miR-146a in AMPK-mediated NAD^+^/SIRT1 activation and their implications in cell senescence and premature aging and aging-related diseases.

## Results

### miRNA screening in senescent and metformin-treated cells

**Fig. 1:**
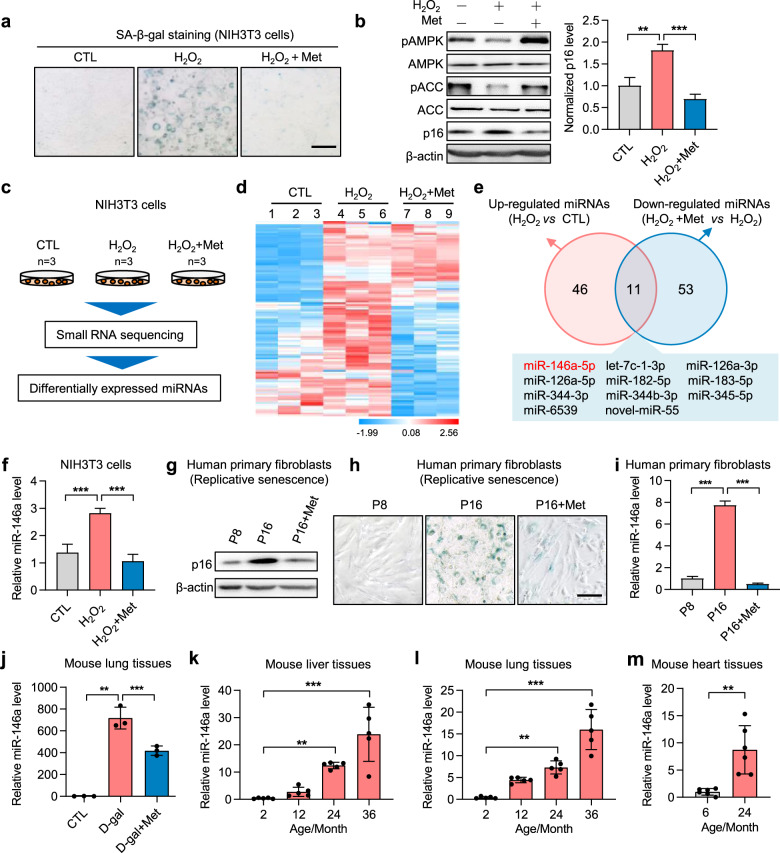
miR-146a is a miRNA differently expressed in senescent cells and metformin-treated cells. **a**, **b** Verification of the anti-senescence effect of metformin in oxidative stress-induced cell senescence. NIH3T3 cells were treated with H_2_O_2_ (400 μM) for 1 h and incubated in a complete medium with or without metformin (Met, 10 mM) for 3 days. **a** Representative images of SA-β-gal staining. Scale bar represents 200 μm. **b** Levels of p-AMPKα1 (pAMPK, Thr172), total AMPKα1, p-ACC (pACC, Ser79), total ACC, p16, and β-actin as detected by immunoblot, with p16 quantification shown (*n* = 5). **c**–**e** Screening of differentially expressed miRNAs increased under H_2_O_2_-induced senescence and decreased after metformin treatment. **c** Experimental diagram of miRNA profiling to screen differentially expressed miRNAs in NIH3T3 cells (*n* = 3). **d** Heatmap of miRNA profiles in NIH3T3 cells. **e** DEseq analysis of differentially expressed miRNAs (threshold fold change ≥1.5 and *p* < 0.05), with the 11 differentially expressed miRNAs shown. **f**–**i** Verification of the increased expression of miR-146a in senescent cells. **f** miR-146a expression in NIH3T3 cells was measured by qRT-PCR (*n* = 5), the cells were treated as described in **a**. **g**, **h** Replicative senescence was induced in human primary sphenoid sinus mucosa fibroblasts by passaging cells. P8, passage 8. **g** The levels of p16 and β-actin were detected by immunoblot. **h** Representative images of SA-β-gal staining in human primary fibroblasts. Scale bar represents 200 μm. **i** miR-146a expression in human primary fibroblasts (*n* = 3). **j** Mice received intraperitoneal (i.p.) injections of D-gal (100 mg/kg/day) daily for 13 weeks to induce progeria, and metformin (100 mg/kg/day, i.p.) was administered to treat mice daily for 13 weeks, and the miR-146a expression was detected in liver tissues (*n* = 3). **k**–**m** Verification of the increased expression of miR-146a in tissues from aged mice. miR-146a expression in liver (*n* = 5), lung (*n* = 5), and heart (*n* = 6) tissues at the indicated months from mice analyzed by qRT-PCR. *p < 0.05, **p < 0.01, ***p < 0.001

We used NIH3T3 cells to establish an oxidative stress-induced senescence model to screen differentially expressed miRNAs in senescent cells and metformin-treated senescent cells. The senescence model was verified by increased senescence-associated β-galactosidase (SA-β-gal) staining (Fig. 1a) and increased p16 levels (Fig. 1b). By contrast, the anti-senescence effect of metformin was confirmed by increased phosphorylation of AMPK and acetyl-CoA carboxylase (ACC), a target protein of AMPK (Fig. 1b), reduced SA-β-gal-positive cells, and decreased p16 levels (Fig. 1a, b). To investigate the potential miRNA involved in the anti-aging effect of AMPK, we performed small-RNA sequencing to explore miRNA-expression profiles (Fig. 1c, d). The levels of 57 miRNAs were increased in senescent cells, whereas 64 miRNAs were decreased following metformin treatment (Fig. 1e). Among these miRNAs, we identified 11 overlapping miRNAs (Fig. 1e and Supplementary Fig. 1). Among them, the expression patterns of miR-146a and miR-126a were confirmed in both stress-induced senescent cells (Fig. 1f and Supplementary Fig. 2) and replicative senescent cells (Fig. 1g–i).

Further, miR-146a was also increased in lung tissues in D-galactose (D-gal)-induced progeria mice compared with paired control mice, but the expression was reduced by metformin treatment (Fig. 1j). The expression of miR-146a and miR-126a were also elevated in the liver, lung, and heart tissues of naturally aged mice (Fig. 1k–m and Supplementary Fig 2b), which was accompanied by the prevalence of aging-related phenotypes, such as organ fibrosis and cardiac remodeling in naturally aged mice (Supplementary Fig. 3). Collectively, these findings suggested that miR-146a and miR-126a may be positively associated with aging or aging-related disease and can be negatively regulated by metformin.

### miR-146a attenuates the effect of metformin on senescence alleviation

**Fig. 2:**
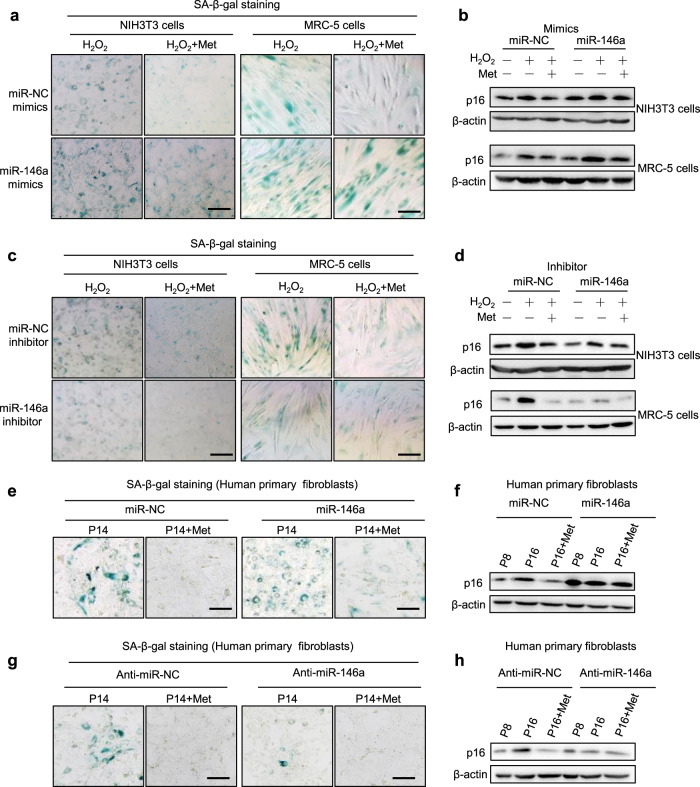
miR-146a attenuates the effect of metformin on the alleviation of senescence. **a**–**d** NIH3T3 or MRC-5 cells were transfected with miR-146a mimics, or miR-146a inhibitors, or respective controls for 24 h, followed by treatment with H_2_O_2_ (400 μM) for 1 h and incubated in complete medium with or without metformin (10 mM) for 3 days (*n* = 5). **a**, **c** miR-146a mimics blunted the suppressive effect of metformin on SA-β-gal-positive cells, whereas miR-146a inhibitors worked reversely in senescent NIH3T3 cells and MRC-5 cells. Scale bars represent 200 μm. **b**, **d** miR-146a mimics impaired the inhibitory effect of metformin on p16 expression, whereas miR-146a inhibitors worked reversely in senescent NIH3T3 cells and MRC-5 cells. **e**–**h** miR-146a regulated replicative senescence in human fibroblasts. The human primary sphenoid sinus mucosa fibroblasts were infected with lentivirus-expressing miR-146a, anti-miR-146a, or the respective controls. Next, replicative senescence was induced by passaging. **e**, **g** miR-146a overexpression blunted the suppressive effect of metformin on SA-β-gal-positive cells, whereas anti-miR-146a worked oppositely in human primary fibroblasts (*n* = 5). Scale bar represents 200 μm. **f**, **h** miR-146a impaired the inhibitory effect of metformin on p16 expression, whereas anti-miR-146a worked oppositely in human primary fibroblasts

To evaluate the effect of miR-146a and miR-126a on AMPK-mediated senescence protection in cells, overexpression and down-expression strategies for these two miRNAs were utilized by using synthetic miRNA mimics and inhibitors (Supplementary Fig. 4a, b). As shown in Fig. 2a, miR-146a mimics increased the percentage of SA-β-gal-positive cells in both NIH3T3 and MRC-5 cells and also attenuated the senescence-inhibitory effect of metformin, similar to the influence of miR-146a mimics on the suppressive effect of metformin on p16 expression (Fig. 2b). In contrast, the miR-146a inhibitor decreased the percentage of SA-β-gal-positive cells and showed a comparable senescence-inhibitory effect as metformin (Fig. 2c). Additionally, miR-146a inhibitor decreased p16 expression and has a similar effect as metformin on p16 expression (Fig. 2d).

The effect of miR-146a on AMPK-mediated senescence protection was also evaluated in replicative senescent cells. To this end, gain-of-function and loss-of-function strategies were used to overexpress or silence miR-146a using lentiviruses (Supplementary Fig. 4c, d). As shown in Fig. 2e, lentivirus-mediated miR-146a overexpression increased the percentage of SA-β-gal-positive cells in replicative senescent cells and attenuated the senescence-inhibitory effects of metformin. miR-146a overexpression also blunted the suppressive effect of metformin on p16 expression (Fig. 2f). Notably, lentivirus-expressing anti-miR-146a showed a senescence-inhibitory effect similar to metformin, as evidenced by SA-β-gal staining and p16 expression (Fig. 2g, h).

We also tested the effect of miR-126a on cellular senescence in NIH3T3 cells. However, neither the miR-126a mimics nor inhibitor had a significant impact on cellular senescence (Supplementary Fig. 5a–d). Taken together, these results indicate that miR-146a, but not miR-126, can attenuate the effect of AMPK on senescence protection. Therefore, we further investigated the role and regulation of miR-146a.

### miR-146a blocks the effect of metformin on aging protection in mice

**Fig. 3:**
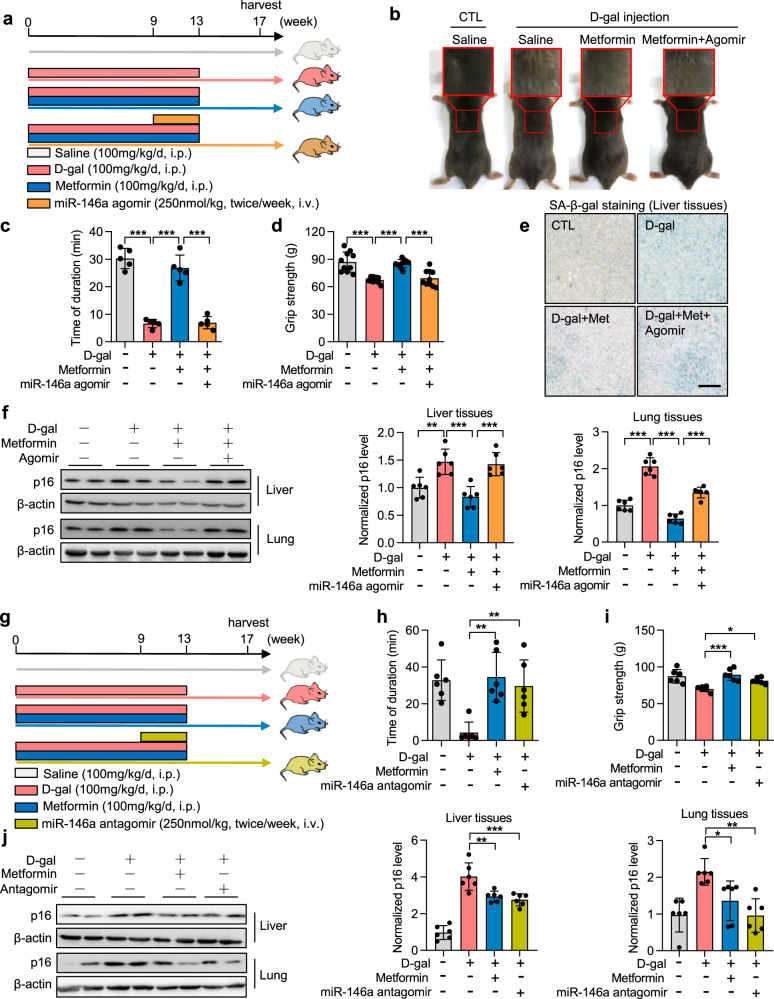
miR-146a blocks the effect of metformin on the alleviation of aging in mice. **a**–**f** The miR-146a agomir attenuates the effect of metformin on aging prevention in a mouse progeria model. **a** Experimental design. Mice received intraperitoneal (i.p.) injection of D-gal (100 mg/kg/day) daily for 13 weeks to induce progeria. Metformin (100 mg/kg/day, i.p.) was administered to treat mice daily for 13 weeks with/without miR-146 agomir treatment. The miR-146a agomir (250 nmol/kg, intravenously [i.v.]) was administered to treat mice twice weekly for 4 weeks. **b** Representative images show that the miR-146a agomir blunted metformin effects on mouse hair phenotype changes. **c**, **d** The miR-146a agomir blunts metformin-induced increases in physical endurance and grip strength in D-gal-induced aged mice. **c** Physical endurance was measured by treadmill, the time before exhaustion was recorded (*n* = 5). **d** Grip strength was measured using a mouse-gripper meter (*n* = 10). **e** The miR-146a agomir inhibits metformin-induced reduction in liver senescence. Representative images of SA-β-gal staining of liver tissues are shown (*n* = 6). Scale bar, 100 μm. **f** The miR-146a agomir inhibits metformin-induced reductions in p16 protein levels in liver and lung tissues (*n* = 6). **g**–**j** The miR-146a antagomir shows comparable anti-aging effects as metformin in a mouse progeria model. **g** Experimental design. Mice were injected with D-gal (100 mg/kg/day, i.p.) daily for 13 weeks to induce progeria. Metformin (100 mg/kg/day, i.p.) was administered to treat mice daily for 13 weeks. The miR-146a antagomir (250 nmol/kg, i.v.) was administered to treat mice twice weekly for 4 weeks. **h**, **i** The miR-146a antagomir shows comparable effects on mouse physical endurance and grip strength as metformin in D-gal-induced aged mice (*n* = 6). **h** Physical endurance was measured by treadmill, and the time before exhaustion was recorded (*n* = 6). **i** Grip strength was measured using a mouse-gripper meter (*n* = 6). **j** The miR-146a antagomir shows comparable effects on p16 levels in liver and lung tissues of D-gal-induced aged mice (*n* = 6). *p < 0.05, **p < 0.01, ***p < 0.001

To determine the effect of miR-146a on AMPK-mediated aging protection in vivo, miR-146a was overexpressed systemically in progeria-like mice by using agomir (the form of miRNA mimics with chemical modification). A progeria-like model was established using chronic D-gal treatment;^26^ metformin and miR-146a agomir were injected later following the procedure shown in Fig. 3a. As expected, D-gal treatment markedly induced progeria-like phenotypes, including increased graying fur of mice and weakened physical power (running endurance and grip force) of mice, whereas metformin played a protective role (Fig. 3b–d). Importantly, consistent with in vitro data, miR-146a agomir impeded the aging protective effect of metformin (Fig. 3b–d). Further analysis showed that miR-146a agomir weakened the suppressive effect of metformin on the SA-β-gal staining and p16 expression, and this phenotype was observed in multiple tissues including the lung, liver, spleen, and kidney (Fig. 3e, f and Supplementary Fig. 6a). Moreover, miR-146a agomir inhibited the repressive effect of metformin on lung fibrosis in progeria mice (Supplementary Fig. 6b).

miR-146a antagomir, which has the reverse sequence for miR-146a agomir, was used to silence miR-146a in vivo (Fig. 3g). Compared with D-gal -treated mice, those treated with the miR-146a antagomir showed greater physical power (Fig. 3h, i). Additionally, miR-146a antagomir reduced p16 expression and the percentage of SA-β-gal-positive cells in the lung, liver, spleen, kidney, and fat of mice (Fig. 3j and Supplementary Fig. 6c, d). Furthermore, the miR-146a antagomir showed a comparable effect on lung fibrosis as metformin in D-gal-induced aged mice (Supplementary Fig. 6e).

Taken together, these results indicated that miR-146a can attenuate the protective effect of metformin on aging and age-related diseases in mice.

### miR-146a reduces the effect of metformin on SIRT1 activation

**Fig. 4:**
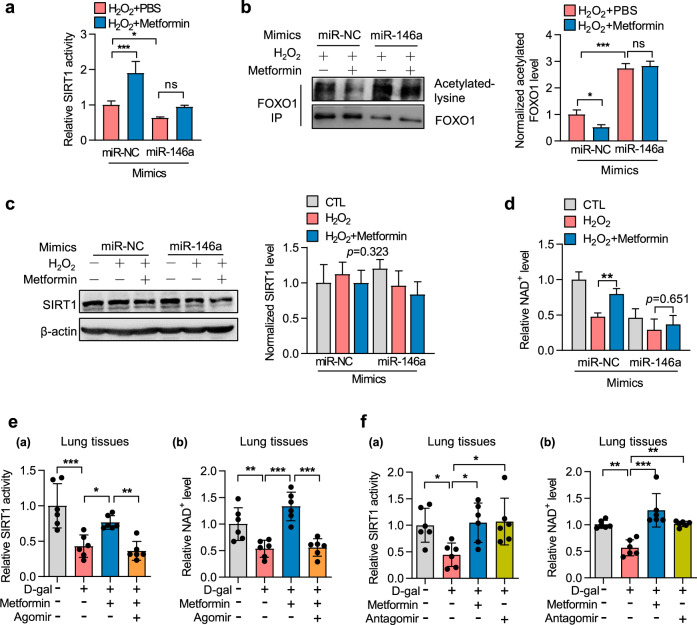
miR-146a reduces the effect of metformin on SIRT1 activity. **a**–**d** miR-146a inhibits the up-regulative effect of metformin on SIRT1 activity in H_2_O_2_-induced senescent NIH3T3 cells. NIH3T3 cells were transfected with miR-NC or miR-146a mimics, followed by H_2_O_2_ and metformin treatment. **a** Enzymatic activity of SIRT1 in cell lysates was measured by the spectroscopic method (*n* = 4). **b** Immunoprecipitation of the SIRT1 target FOXO1 from cells, followed by the analysis of total and acetylated FOXO1 levels and the ratio of acetylated to FOXO1 levels is shown (*n* = 3). **c** SIRT1 protein expression was tested and representative immunoblots are shown (*n* = 4). **d** Intracellular NAD^+^ level was assayed by spectroscopic method (*n* = 4). **e**, **f** miR-146a agomir inhibits the up-regulative effect of metformin on SIRT1 activity and NAD^+^ levels, whereas the miR-146a antagomir shows comparable effects to those of metformin on D-gal-induced aged mice. SIRT1 activity and NAD^+^ levels in lungs from mice receiving D-gal, metformin, and the miR-146a agomir (**e**) or antagomir (**f**) were determined (*n* = 6). *p < 0.05, **p < 0.01, ***p < 0.001. ns means no significance

Several studies have shown that metformin is an AMPK activator^27^ and that AMPK’s anti-aging effect is closely related to its positive relationship with SIRT1.^14,16,17^ We investigated whether the functions of miR-146a in cellular senescence and mouse aging were associated with SIRT1 activity. Our results showed that metformin improved SIRT1 activity in senescent cells, whereas miR-146a overexpression reduced SIRT1 activity and impaired the effect of metformin on SIRT1 activity restoration (Fig. 4a). A similar result was obtained by monitoring FOXO1 acetylation,^28^ a sign of SIRT1 activity (Fig. 4b). Surprisingly, SIRT1 protein levels were unchanged (Fig. 4c). Next, we assessed whether miR-146a can affect the cellular level of NAD^+^, the sole co-enzyme of SIRT proteins. The results showed that miR-146a mimics reduced NAD^+^ levels in senescent cells and attenuated the effect of metformin on NAD^+^ level restoration (Fig. 4d). Given that SIRT1 plays an important role in mitochondrial function,^7,9^ we tested the impact of miR-146a on mitochondrial function. miR-146a reduced the protective effect of metformin on mitochondrial function, including ATP level, mitochondrial membrane potential, genes controlling mitochondria biogenesis, and mitochondrial content (Supplementary Fig. 7).

Next, the effect of miR-146a on AMPK-mediated SIRT1 activity and NAD^+^ level was evaluated in vivo using the D-gal-induced progeria mouse model. Like their influence on aging phenotypes described in Fig. 3, miR-146a agomir impeded the rescue effect of metformin on NAD^+^ level and SIRT1 activity (Fig. 4e). By contrast, miR-146a antagomir showed a similar role as metformin in recovering NAD^+^ level and SIRT1 activity in progeria mice (Fig. 4f).

These results suggested that miR-146a was involved in the mechanism underlying the metformin-mediated anti-aging effect partially via the NAD^+^/SIRT1 pathway.

### miR-146a directly targets the *Nampt* gene and decreases NAD^+^ synthesis

**Fig. 5:**
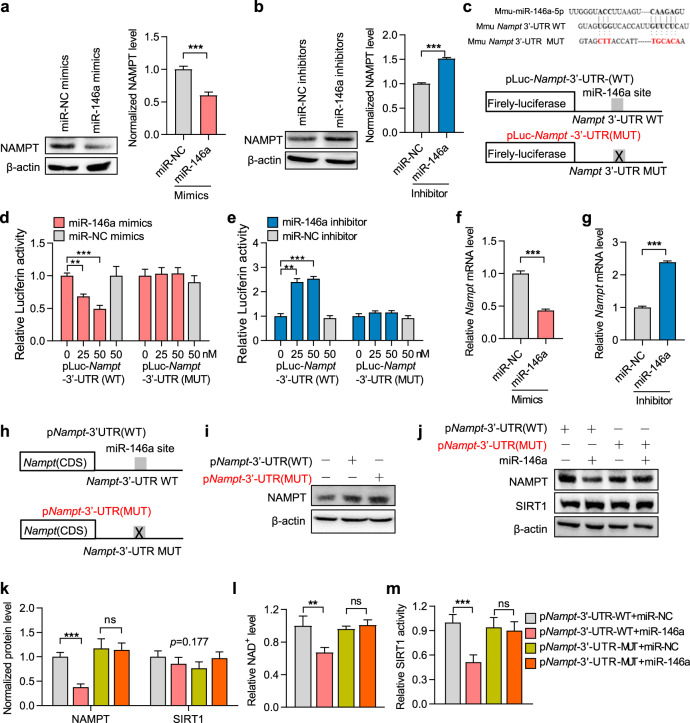
miR-146a decreases SIRT1 activity by directly targeting *Nampt* mRNA. **a**–**g** miR-146a directly targets the *Nampt* 3′-UTR. The NAMPT protein level in NIH3T3 cells transfected with miR-146a mimics (**a**) or inhibitors (**b**), with relative fold changes are shown (*n* = 3). **c** The mouse miR-146a sequence and the predicted miR-146a target site in the 3′-UTR of WT and MUT *Nampt* (upper). Schematic of the luciferase reporter plasmids with the WT or MUT *Nampt* 3′-UTRs (lower). **d**, **e** Relative luciferase activity in HEK293T cells transfected with the indicated luciferase reporter plasmids along with miR-146a mimics or inhibitors, respectively (*n* = 3). **f**, **g** Endogenous *Nampt* mRNA levels in NIH3T3 cells transfected with miR-146a mimics or inhibitor (*n* = 3). **h**–**m** miR-146a decreases SIRT1 activity by directly targeting the *Nampt* 3′-UTR to inhibit NAMPT expression and NAD^+^ level. **h** Schematic of plasmids *Nampt* 3′-UTR containing wild-type or mutated miR-146a targeting site. **i** NAMPT protein level in NIH3T3 cells transfected with the indicated plasmids. **j**, **k** NAMPT and SIRT1 expression in NIH3T3 cells transfected with miR-146a mimics and the indicated plasmids, with relative fold changes shown (*n* = 3). **l**, **m** NAD^+^ level and SIRT1 activity in NIH3T3 cells transfected with miR-146a mimics and the indicated plasmids (*n* = 3). **p < 0.01, ***p < 0.001. ns means no significance

Following on, we wondered whether miR-146a can target NAMPT, a rate-limiting enzyme of NAD^+^ synthesis in the salvage pathway, given that NAMPT expression is downregulated in senescent cells but can be restored by metformin.^16^ In this study, we verified that NAMPT expression was reduced in senescent cells and tissues of aged mice and was increased after metformin treatment (Supplementary Fig. 8). Additionally, miR-146a overexpression reduced the effect of metformin on NAMPT expression in lung tissues of aged mice (Supplementary Fig. 9). Furthermore, miR-146a also decreased the protein expression of NAMPT in mouse and human proliferative NIH3T3 and MRC-5 cells (Fig. 5a, b and Supplementary Fig. 10a, b). According to bioinformatics analysis using the miRDR, MiRWalk 2.0, and TargetScanMouse software, miR-146a may target the 3′-untranslated region (UTR) of the human *NAMPT* and mouse *Nampt* mRNAs (Fig. 5c and Supplementary Fig. 10c). Next, we determined whether *Nampt* mRNA may be directly targeted by miR-146a. Using luciferase reporter genes with the wild-type (WT) sequence or mutations (MUT) for the miR-146a targeting site located on the *Nampt* 3′-UTR (Fig. 5c), we found that miR-146a mimics inhibited the activity of the WT reporter but not that of the MUT reporter (Fig. 5d). Conversely, miR-146a inhibitor increased the WT reporter activity but not that of the MUT reporter (Fig. 5e). Coincidently, miR-146a overexpression and inhibition decreased and increased the *Nampt* mRNA level, respectively (Fig. 5f, g and Supplementary Fig. 10d–g). These results support the mechanism that miR-146a can directly target the *Nampt* 3′-UTR and negatively regulate *Nampt* expression.

As multiple genes are potential targets of miR-146a, we performed further experiments to clarify whether miR-146a affects NAD^+^ levels and SIRT1 activity by directly targeting the *Nampt* 3′-UTR. We generated *Nampt*-expression plasmids containing the same coding region but different 3′-UTRs, namely p*Nampt*-3′-UTR(WT) or p*Nampt*-3′-UTR(MUT) (Fig. 5h), and verified that the transfection of WT or MUT alone both increased NAMPT protein expression (Fig. 5i). However, co-transfection with miR-146a mimics resulted in decreased NAMPT expression in p*Nampt*-3′-UTR(WT) but not p*Nampt*-3′-UTR(MUT) transfected cells (Fig. 5j, k). Consistently, the suppressive effects of the miR-146a mimics on NAD^+^ levels and SIRT1 activity were impaired in p*Nampt*-3′-UTR(MUT)-transfected cells alone (Fig. 5l, m). These results demonstrate that miR-146a inhibits NAMPT/NAD^+^ levels and SIRT1 activity by directly targeting *Nampt* 3′-UTR.

### Metformin downregulates miR-146a transcription in an AMPK-NF-κB-dependent manner

**Fig. 6:**
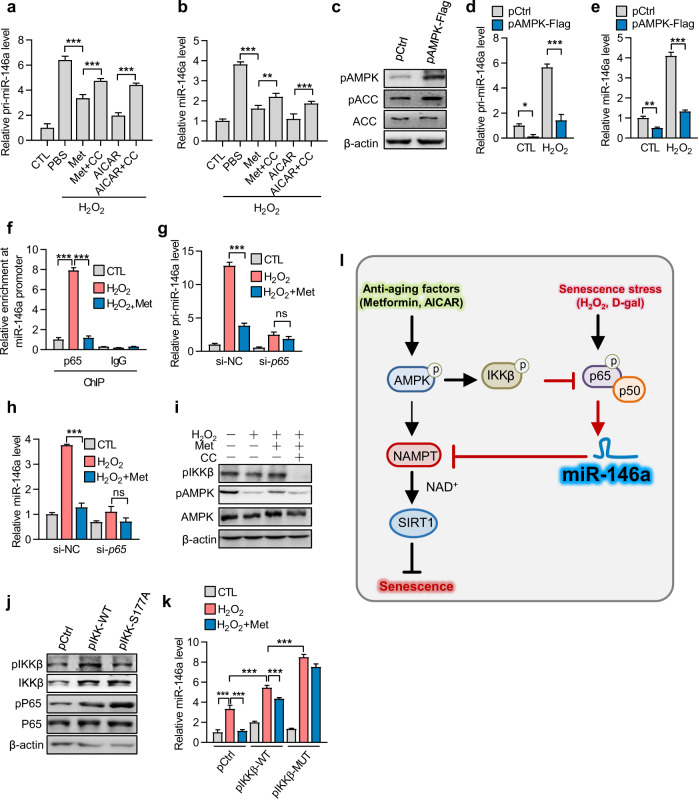
Metformin regulates miR-146a transcription in an AMPK-NF-κB-dependent manner. **a**, **b** Inhibition of AMPK promotes pri-miR-146a and miR-146a expression in senescent cells. NIH3T3 cells were treated with H_2_O_2_ and incubated in complete medium with or without treatment with the AMPK activators metformin (10 μM), AICAR (10 μM), alone or plus AMPK inhibitor Compound C (CC, 10 μM) for 3 days, then the levels of pri-miR-146a (**a**) and miR-146a (**b**) were tested (*n* = 3). **c**–**e** Overexpression of AMPK reduces pri-miR-146a and miR-146a expression in NIH3T3 cells. **c** Overexpression of AMPK in NIH3T3 cells. Immunoblots were performed to evaluate levels of p-AMPKα1, p-ACC, ACC, and β-actin in NIH3T3 cells transfected with pAMPKα1-flag or a control plasmid (pCtrl). **d**, **e** NIH3T3 cells were transfected with pAMPKα1-flag or pCtrl for 24 h, followed by treatment with H_2_O_2_ and incubation in a complete medium for 3 days. Levels of pri-miR-146a (**d**) and miR-146a (**e**) were evaluated (*n* = 3). **f**–**h** AMPK inhibits miR-146a expression by reducing p65 binding to the miR-146a promoter. **f** ChIP assays were performed to analyze p65 binding to the miR-146a promoter in NIH3T3 cells treated with H_2_O_2_ and metformin (*n* = 3). Following transfection with *sip65* or negative control siRNA (si-NC), NIH3T3 cells were treated with H_2_O_2_ and metformin for 3 days, and pri-miR-146a (**g**) and miR-146a (**h**) levels were evaluated (*n* = 3). **i**–**k** AMPK inhibits miR-146a expression via IKKβ-mediated suppression of the NF-κB pathway. **i** Levels of p-ACC, p-AMPK, and p-IKKβ (Ser177) in NIH3T3 cells were tested by immunoblot. **j** Levels of p-IKKβ, IKKβ, p-p65 (Ser536), and p65 in NIH3T3 cells transfected with the pIKKβ-WT or pIKKβ-S177A (pIKKβ-MUT) plasmid. **k** Metformin regulates miR-146a level in an IKKβ-dependent manner. NIH3T3 cells were transfected with pIKKβ-WT and pIKKβ-S177A (pIKKβ-MUT) and then treated with H_2_O_2_ and metformin, followed by evaluation of miR-146a expression (*n* = 3). **l** Schematic depicting the miRNA-specific mechanism related to metformin-mediated prevention of senescence. Oxidative stress increased the expression of miR-146a, which targets the *Nampt* 3’-UTR to reduce NAMPT and NAD^+^ levels and SIRT1 activity, ultimately leading to cell senescence. Metformin induces AMPK activation to repress miR-146a transcription in an NF-κB-dependent manner, which increases NAMPT/NAD^+^ levels and SIRT1 activity and consequently suppresses cell senescence and mouse aging. *p < 0.05, **p < 0.01, ***p < 0.001. ns means no significance

Next, we evaluated how metformin suppresses the expression of miR-146a. We first compared the alteration of pri-miR-146a and mature miR-146a levels to determine whether metformin regulates miR-146a expression at the transcriptional level. The results showed that the same alteration pattern was observed in both pri-miR-146a and mature miR-146a (Fig. 6a, b), indicating that metformin suppressed *Mir146a* gene expression at the transcriptional level. To verify that the action of metformin is related to AMPK activation, another AMPK activator AICAR and the AMPK inhibitor compound C (CC) were used. Similar to metformin, AICAR decreased the levels of both pri-miR-146a and mature miR-146a levels, whereas CC eliminated the suppressive effect of AICAR and metformin (Fig. 6a, b). Additionally, we analyzed the effect of AMPK overexpression on miR-146 expression (Fig. 6c). Consistent with the pharmacological approach, AMPK overexpression decreased the levels of pri-miR-146a and mature miR-146a (Fig. 6d, e). These results demonstrate that AMPK inhibited miR-146a expression at the transcriptional level.

Then, the AMPK-affected miR-146a transcription signal was investigated. Based on preliminary tests and literature research, the investigation was finally focused on the NF-κB pathway.^29^ Chromatin immunoprecipitation (ChIP) assay results showed that the p65 subunit of NF-κB bound to the promoter region of the *Mir146a* gene, and metformin weakened the binding (Fig. 6f). Moreover, small-interfering RNA-mediated p65 knock-down (*sip65*) reduced the levels of pri-miR-146a and mature miR-146a and impaired the suppressive effect of metformin on miR-146a transcription (Fig. 6g, h). These results clarify that the NF-κB activity, at least in part, contributed to the *Mir146a* transcription and that metformin can suppress this role.

Finally, we assessed the relationship between the NF-κB pathway and AMPK. The results showed that metformin increased the phosphorylation (S177) of endogenous IκB kinase β (IKKβ), whereas CC decreased IKKβ phosphorylation (Fig. 6i). The significance of IKKβ phosphorylation on miR-146a levels was also analyzed using a variant of IKKβ with the phosphorylation site mutated (S177A), which resulted in the increased phosphorylation of p65 (Fig. 6j). This IKKβ mutant increased the level of miR-146a, and impaired the suppressive effect of metformin on miR-146a levels (Fig. 6k).

Taken together, these results suggest that metformin reduces miR-146a expression by promoting IKKβ phosphorylation, which suppressed p65 phosphorylation and subsequently miR-146a transcription.

## Discussion

In this study, we identified miR-146a as a senescence-associated miRNA and showed its correlation with the AMPK pathway. We found that the miR-146a level was increased in senescent cells and decreased in metformin-treated cells, respectively. Similar results were observed in aged mice and mice with age-related diseases. Importantly, we showed that miR-146a can impair AMPK-mediated improvement of aging prevention, SIRT1 activity, and NAD^+^ levels by directly targeting the 3′-UTR of *Nampt*. Moreover, we showed that AMPK suppressed the transcription of *Mir146a*, which is dependent on the activity of NF-κB signaling. Therefore, these results suggest that AMPK upregulates *Nampt* expression and NAD^+^ levels via repressing miR-146a, which affects SIRT enzyme-driven anti-aging processes. Furthermore, these findings further elucidate the interplay between AMPK signaling and miRNA-mediated *Nampt* expression and downstream anti-aging processes (Fig. 6l).

Cellular NAD^+^ levels significantly affect the activity of enzymes that play important roles in aging defense, such as SIRT family deacetylases and poly(ADP-ribose) polymerases.^6,17^ Previous studies confirmed that NAD^+^ levels declined with biological aging, and the elevation of NAD^+^ levels by genetic or pharmacologic means can prolong lifespan.^6^ NAD^+^ synthesis is regulated by two pathways: the salvage and *de novo* pathways. The salvage pathway is sensitive to metabolic changes and impacts several physical and pathogenic processes, with NAMPT identified as one of the rate-limiting enzymes of this pathway.^30^ Previous studies revealed that AMPK activation can increase intracellular NAD^+^ levels by positively regulating NAMPT expression in senescent cells, and that NAMPT inhibitors can weaken the anti-aging effects of AMPK.^16,31^ Although the expression of *Nampt* at the transcriptional level has been elucidated,^18,20,21^ we identified new miRNA-mediated mechanisms that regulate *Nampt* expression. The gene encoding NAMPT can be targeted by miR-374a and miR-568 in humans,^22^ and miR-34a and miR-26b in mice.^23^ Among them, miR-34a is the only miRNA implicated in aging.^32^ However, its association with AMPK remains unknown. Because miR-374a and miR-34a can target *NAMPT*, it would be interesting to test whether these miRNAs participate in cell senescence. To establish a connection between miRNA-mediated suppression and AMPK activation in senescence, we used small-RNA sequencing and acquired several miRNAs with level alterations occurring oppositely in cellular senescence and AMPK activation (metformin treatment). This miRNA screening and subsequent experimental verification identified miR-146a as a potential metformin-responsible pro-aging miRNA in human and mouse senescent cells in vitro and in a progeria mouse model in vivo.

Our findings support the idea that miR-146a promotes aging. We found that miR-146a directly targeted *Nampt*, thereby downregulating NAD^+^ levels in cells and subsequently suppressing SIRT-mediated anti-aging signaling. This is consistent with previous studies on the role of miR-146a in aging. Deng and colleagues demonstrated that miR-146a induces lineage-negative bone marrow cell senescence by targeting the gene encoding polo-like kinase 2.^33^ However, miR-146a-deficient mice showed amelioration of the inflammatory bone marrow microenvironment and promoted hematopoietic stem cell inflammation by targeting interleukin-6 and tumor necrosis factor (TNF)-α,^34,35^ while also protecting against ovariectomy-induced bone loss during aging by targeting *Wnt1* and *Wnt5a*,^36^ suggesting that miR-146a functions as an age-related inflammation suppressor. In this study, miR-146a was positively correlated with cellular senescence both in vitro and in vivo. Collectively, miR-146a might function as a pro-aging factor, at least in oxidative stress-induced aging.

Given that the expression alterations of miR-146a and pri-miR-146a were the same, we think that either oxidative stress or AMPK activation-mediated regulation of miR-146a occurs at the transcriptional level. According to previous studies, p65 transcription factors are involved in the regulation of the *Mir146a* gene, which acts as an activator.^37^ Our data based on multiple assays confirmed the promotive role of p65 and the connected NF-κB signaling in the transcription of the *Mir146a* gene. Further, we verified that AMPK-mediated regulation of miR-146a is largely dependent on the phosphorylation of IKKβ, a p65 inhibitor.^38^ These results may offer a novel insight into the mechanism of AMPK-mediated *Nampt* expression and NAD^+^ levels.

This study has some limitations. First, miR-146a was initially identified as a pro-aging miRNA that can be suppressed by metformin, and its connection with AMPK might not be exclusive, as metformin does not directly target AMPK. Herein, although our data using AICAR, AMPK expression plasmid, and CC provided more evidence for the effect of AMPK on miR-146a expression, AMPK-independent miR-146a regulation following metformin treatment cannot be completed ruled out. Second, we could not clarify the involvement of other *NAMPT*-targeting miRNAs, such as miR-34a and miR-374a,^22,23^ in AMPK-mediated NAMPT expression. Further studies involving these miRNAs in the AMPK pathway would enrich our understanding of miRNA biology and metabolic regulation of aging. Third, we did not evaluate other possible roles of miR-146a relevant to aging, such as targeting of NF-κB or TNF receptor-associated factor 6,^39–42^ which may result in anti-aging effects. Finally, our in vivo study only used agomir and antagomir to evaluate miR-146a function in aged mice. Further studies using miR-146a-knockout mice would provide more solid evidence to elucidate the role of miRNA-146a in natural aging and age-related diseases.

In conclusion, miR-146a affects *Nampt* expression and can be regulated by AMPK in vitro and in vivo. This study also demonstrated a new mechanism underlying the regulation of AMPK in NAMPT expression, which is characterized by AMPK-mediated IKKβ phosphorylation, NF-κB inhibition, NF-κB-mediated *Mir146a* transcription, and miR-146a-induced NAMPT suppression. Together with the data involving changes in NAD^+^ levels and SIRT1 activity, this study expands the understanding of miRNA-related mechanisms involved in the aging process and suggests that miR-146a may be of value for slowing aging and treating aging-related diseases.

## Materials and methods

### Reagents and antibodies

Metformin was purchased from Sangon Biotech (Shanghai, China), D-(+)-Galactose (D-gal was purchased from Sigma-Aldrich (St. Louis, MO, USA), and CC and AICAR were purchased from Selleck Chemicals (Houston, TX, USA). Antibodies against NAMPT (ab45890) and CDKN2A/p16INK4a (ab51243) were obtained from Abcam (Cambridge, UK). Antibodies against phosphorylated (p)-AMPKα (Thr172) (40H9), AMPKα (D5A2), p-Acetyl-CoA carboxylase (Ser79) (3661), acetylated lysine (9441), p-IKKα/β (Ser176/180) (16A6), IKKβ (D30C6), p-NF-κB p65 (Ser536) (93H1), and NF-κB p65 (D14E2) were obtained from Cell Signaling Technology (Danvers, MA, USA). Antibodies against FOXO1 (ET1608-25) and SIRT1 (ER130811) were obtained from Huabio (Hangzhou, China), and that against β-actin (bs-10966R) was obtained from Bioss Antibodies (Beijing, China). The miR-146a-5p mimics (sense, 5′-UGAGAACUGAAUUCCAUGGGUU-3′ and anti-sense, 5′-CCCAUGGAAUUCAGUUCUCAUU-3′), miR-146a-5p inhibitor (5′-AACCCAUGGAAUUCAGUUCUCA-3′), scrambled control miRNA (miR-NC), miR-146a-5p agomir (sense, 5′-UGAGAACUGAAUUCCAUGGGUU-3′ and anti-sense, 5′-CCCAUGGAAUUCAGUUCUCAUU-3′, miR-146a-5p antagomir (5′-AACCCAUGGAAUUAGUUCUCA-3′), and primers for detecting miR-146a were purchased from GeneCopoeia (Guangzhou, China). These modified miRNA agomir/antagomir have a high affinity to cell membranes and higher stability and effect in both in vitro and in vivo experiments.^43,44^

### Cell culture and treatments

Murine fibroblast NIH3T3 cells, human embryonic lung fibroblast MRC-5 cells, and human embryonic kidney cells (HEK293) were obtained from the Shanghai Institutes for Biological Sciences of the Chinese Academy of Sciences (Shanghai, China). Human primary fibroblasts were obtained from the sphenoid sinus mucosa tissues of 40-60 individuals. All cells were cultured in Dulbecco’s modified Eagle medium (DMEM) supplemented with 10% fetal bovine serum (FBS) and maintained in a humidified atmosphere at 37 °C with 5% CO_2_. For senescence induction, we used a modified H_2_O_2_-treatment protocol. Briefly, NIH3T3 cells were trypsinized and suspended in phosphate-buffered saline (PBS) at a density of 1 × 10^6^ cells/ml. The suspension was then transferred to an Eppendorf tube, and the cells were exposed to 400 μM H_2_O_2_ and incubated at 37 °C for 45 min. During H_2_O_2_ treatment, the tube was gently turned upside down every 5 to 10 min. H_2_O_2_ treatment was terminated by centrifugation for 5 min at 800 rpm, the supernatant was removed, and cells were resuspended in a complete medium (DMEM with 10% FBS). The treated cells were then seeded onto a plate for the indicated experiments, followed by exposure to different treatments, as described in the figure legends and our previous studies.^45^ For replicative senescence induction, human sphenoid sinus mucosa fibroblasts were split 1:3 for passaging until they reached senescent growth arrest and replicative senescence. As reported, primary fibroblasts from 59 aged human skin tissues induced replicative senescence at passage number 16,^46^ and we established replicative senescence at passage numbers 14 or 16.

### Cell transfection

For overexpression of miR-146a mimics or inhibitors, NIH3T3, MRC-5 cells, or HEK293 cells were transfected with RNA at a final concentration of 50 nM using Jet PRIME transfection reagents (PolyPlus, Illkirch, France) according to the manufacturer’s instructions. Negative control mimics or inhibitors (GeneCopoeia) were transfected to serve as matched controls. For overexpression of *NAMPT* with its 3′-UTR, cDNA encoding NAMPT with the WT or MUT 3′-UTR was amplified and inserted into pZX-FR01 (GeneCopoeia), followed by transfection using Jet PRIME transfection reagents (PolyPlus) into NIH3T3 cells according to the manufacturer’s instructions.

### SA-β-gal staining

Cellular SA-β-gal activity was assayed using an SA-β-gal staining kit (C0602; Beyotime, Beijing, China) according to the manufacturer’s instructions. Senescent cells were identified as bluish-green-stained cells using microscopy, as previously describled.^45^

### Measurement of NAD^+^ level and SIRT1 activity

NAD^+^ levels in cells and animal tissues were measured using an NAD^+^/NADH quantification kit (NAD-2-Y; Suzhou Comin Biotechnology, Suzhou, China) according to the manufacturer’s instructions. Values were normalized according to protein concentration. SIRT1 activity was measured using the universal SIRT1 activity assay kit (ab156915; Abcam) according to the manufacturer’s instructions.

### ATP level quantitation

Intracellular ATP level assay was performed using an ATP assay kit (A095-1-1, Nanjing Jiancheng Biotechnology Institute, Nanjing, China) as per the manufacturer’s protocol. Values were normalized based on the protein concentration.

### Mitochondrial membrane potential assay (JC-1)

Mitochondrial membrane potential was detected by a mitochondrial-specific dual fluorescence probe, JC-1(40705ES03, YEASEN Biotech Co., Ltd, Shanghai, China). Cells were incubated with JC-15 μg/ml at 37 °C for 30 min, and subsequently, the cells were washed twice with PBS and imaged using a fluorescence microscope. Next, cells were collected using trypsin/EDTA and resuspended in PBS. Fluorescence intensity was detected using excitation and emission wavelengths of 550 and 600 nm, and with excitation and emission wavelengths of 585 and 535 nm, respectively, and the values were corrected and normalized for the excitation wavelengths of A550 nm/A485 nm.

### Mitochondrial content assay

Mitochondrial content was monitored using the MitoTracker Green probe (40742ES50, YEASEN Biotech Co., Ltd, Shanghai, China). Cells were incubated with MitoTracker Green at 37 °C for 30 min, and the cells were washed twice with PBS and imaged under a fluorescence microscope. Cells were collected using trypsin/EDTA and resuspended in PBS. Fluorescence intensity was detected with excitation and emission wavelengths of 490 and 516 nm, respectively, and the values were normalized according to protein concentration.

### Plasmids and DNA transfection

Vectors containing cDNA of AMPKα1 were obtained by amplifying mouse cDNA and insertion into the pcDNA3.1 expression vector (pAMPK-Flag). Endotoxin-free plasmids were transfected using the Jet PRIME transfection reagent (PolyPlus) according to manufacturer instructions.

### Lentivirus packaging and cell-line screening

Vectors containing the cDNA of WT IKKβ were obtained by amplifying mouse cDNA with Phanta® Super-Fidelity DNA Polymerase (cat. No. P511-01; Vazyme Biotech co., ltd, Nanjing, China). The IKKβ S177A mutant was obtained by amplifying the cDNA of WT IKKβ, followed by insertion into a pLVX-IRES-ZsPuro lentiviral expression vector (Clontech, Shiga, Japan). Lentivirus was produced by co-transfection of lentivirus packing plasmids with psPAX2 and pMD2.G using the Jet PRIME transfection reagents (PolyPlus) into HEK293 cells according to the manufacturer’s instructions. The medium was changed after 24 h, and the medium containing the virus was collected after another 72 h, followed by 10 min of centrifugation at 2000 × *g* and the supernatant was collected and stored at −80 °C. The supernatant was used to infect NIH3T3 cells with 10 μg/ml polybrene (cat. No. H9268; Sigma-Aldrich), and resistant colonies were selected after 8 h using 50 μg/ml puromycin (cat. No. 58-58-2; Sangon Biotech). miR-146a and anti-miR-146a vectors were purchased from GeneCopoeia (Guangzhou, China), and the miR-146a and anti-miR-146a lentivirus were obtained by co-transfection of lentivirus packing plasmids with psPAX2 and pMD2.G using the Jet PRIME transfection reagents (PolyPlus) into HEK293 cells according to the manufacturer’s instructions as described above.

### Luciferase reporter constructs and dual-luciferase reporter assay

HEK293 cells were trypsinized and seeded into 24-well plates before the day of transfection to achieve ~80% confluence before transfection. A corresponding NAMPT-luciferase reporter (pZX-FR01-NAMPT 3′-UTR) containing miR-146a-5p-binding sites was co-transfected with mimics or inhibitors of miR-146a-5p, and miR-NC, respectively, into HEK293 cells, using the Jet PRIME transfection reagent (PolyPlus). The cells were harvested at 48 h after transfection, the lysates were extracted, and luciferase activity was measured using a dual-luciferase reporter assay (Promega, Madison, WI, USA). The results were expressed as relative luciferase activity (Firefly Luc/Renilla Luc).

### RNA interference (RNAi)

*sip65* (sense, 5′-GCAUGCGAUUCCGCUAUAATT-3′ and anti-sense, 5′-UUAUAGCGGAAUCG CAUGCTT-3′) was obtained from Sangon Biotech (Shanghai, China). For RNAi experiments, cells were seeded into 6-wells plates and transfected with siRNA using Jet PRIME transfection reagent (PolyPlus), as described previously.^47^ The medium was replaced with complete DMEM 24 h after transfection, and the cells were used for subsequent experiments.

### Measurement of mRNA and miRNA

Total RNA was extracted from cultured cells or tissues using Trizol reagent (Takara, Shiga, Japan), and first-strand cDNA was synthesized using HiScript II Q RT SuperMix for qPCR (+gDNA wiper) (cat. No. R223-01; Vazyme Biotech co., ltd, Nanjing, China). Quantitative (q)PCR was performed using SYBR Green qPCR master mix (cat. No. B21203; BioTools Houston, TX, USA), and the relative amount of cDNA was calculated by the comparative CT method using the 18S ribosomal RNA sequence as a control. For miRNA, 1 μg total RNA was reverse transcribed using the All-in-One miRNA qRT-PCR detection kit (GeneCopoeia) according to manufacturer instructions. U6 RNA was used as a housekeeping control. The primer sequences used for qPCR amplification are listed in Supplementary Table S1.

### Chromatin immunoprecipitation assay (ChIP) and ChIP-qPCR

A ChIP assay was performed to determine the binding of NF-κB p65 to the miR-146a promoter. NIH3T3 cells were cross-linked by adding formaldehyde directly to the medium to a final concentration of 1% and incubated for 10 min at room temperature. and the cross-linking reaction was stopped by adding glycine (up to 0.125 M) for 5 min. Ice-cold PBS was then added, the cells were washed, and samples were collected by scraping. After centrifugation, the cells were lysed in lysis buffer [(1% sodium dodecyl sulfate (SDS), 10 mM EDTA, and 50 mM Tris-HCl (pH 8.1)] containing a protease inhibitor cocktail (cat. No. B14002; BioTools). Chromatin was sheared by sonication (ultrasound sonicator, JY92-IIN; Xinyi, Ningbo, China), resulting in DNA fragments of between 200 bp and 800 bp. After centrifugation to remove cell debris, 5% of the sample was kept as input, and the remaining sample was diluted 10-fold with dilution buffer [0.01% SDS, 1.1% Triton X-100, 1.2 mM EDTA, 16.7 mM Tris-HCl (pH 8.1), and 167 mM NaCl] containing a protease inhibitor cocktail. Next, with protein A/G agarose 50% slurry (Selleck Chemicals) at 4 °C for pre-clearing. IP analysis was performed at 4 °C overnight using anti-NF-κB p65 (D14E2; 1:200; Cell Signaling Technology), with anti-rabbit IgG used as a control for nonspecific binding. Immune complexes were collected with the protein A/G agarose 50% slurry over 4 h at 4 °C, after which the agarose beads were washed for 5 min on a rotating platform at 4 °C using 1 ml of each of the following buffers and centrifugation at 500 × *g* for 4 min at 4 °C: one wash with low-ionic strength buffer [0.1% SDS, 1% Triton X-100, 2 mM EDTA, 20 mM Tris-HCl (pH 8.1), and 150 mM NaCl], one wash with high-salt wash buffer [0.1% SDS, 1 % Triton X-100, 2 mM EDTA, 20 mM Tris-HCl (pH 8.1), and 150 mM NaCl], one wash with LiCl wash buffer [0.25 M LiCl, 1% NP40, 1% deoxycholate, 1 mM EDTA, and 10 mM Tris-HCl (pH 8.1)], and two washes with TE buffer [0.25 M LiCl, 1% NP40, 1% deoxycholate, 1 mM EDTA, and 10 mM Tris-HCl (pH 8.1)]. DNA fragments were collected and purified using a FastPure Gel DNA extraction mini kit (DC301; Vazyme, Nanjing, China) in 30 μl of EB (elution buffer). Then, immunoprecipitated DNA was analyzed by qPCR using promoter-specific primer pairs for NF-κB-binding sites in the miR-146a promoter (Supplementary Table S2). The negative-control primers flanking a region-specific for the glyceraldehyde 3-phosphate dehydrogenase gene were used to represent the specificity of the ChIP reactions. DNA purified from the sonicated lysate was analyzed by qPCR using the same primer sets, which were used as input controls. The expression of a target DNA sequence was normalized to the input DNA and represented as fold enrichment relative to the control (set as one-fold).^48^

### Immunoblot analysis

Immunoblot analysis was performed as previously described.^49^ Briefly, cells were lysed in RIPA buffer containing a protease inhibitor cocktail (cat. No. B14002; BioTools) for 30 min, followed by centrifugation for 10 min, and 50 μg of total proteins were loaded onto an SDS-polyacrylamide gel and separated by electrophoresis, followed by transfer to PVDF membrane (cat. No. GVWP2932A; Millipore, Billerica, MA, USA). Target proteins were probed with the corresponding primary antibodies under optimized conditions and then incubated with the secondary antibody. Immunological signals were surveyed using an Immobile western chemiluminescence HRP substrate kit (cat. No. 501926; Zen Bioscience, Durham, NC, USA) and the Fusion Solo imaging system (VIBER LOURMAT, Marne-la-Vallée, France).

### Small-RNA sequencing and data analysis

Small-RNA sequencing was conducted by Novogene (Beijing, China), and miRNA data analysis of the control, H_2_O_2_, and H_2_O_2_ plus metformin groups was performed by Chengdu Bayer Biotechnology Corporation (Chengdu, China). Small-RNA sequencing was performed in triplicate using three independent sets of RNA preparations. DESeq analysis was used to identify differentially expressed miRNAs with a threshold of fold change ≥1.5 and a *p* < 0.05.

### Mice

Six-to-eight-week-old male C57BL/6J mice were purchased from the DaShuo Biotechnology Company (Chengdu, China). Mice were kept in a pathogen-free facility at the Experimental Center of West China Hospital and fed ad libitum, and were kept on a standard 12-h light/dark cycle. The protocols for the animal experiments were approved by the Laboratory Animal Ethics Committee of West China Hospital, Sichuan University.

### Animal experiments

For experiments assessing the effect of the miR-146a agomir on AMPK activity, mice were randomly divided into four groups: control, D-gal, D-gal plus metformin, and D-gal plus Metformin and miR-146a agomir. Mice in the control, D-gal, and D-gal plus metformin groups were treated with a miR-146a agomir negative control.

For experiments assessing the effect of the miR-146a antagomir on aging, mice were randomly divided into four experimental groups: control, D-gal, D-gal plus metformin, and D-gal plus miR-146a antagomir. Mice in the control, D-gal, and D-gal plus metformin groups were treated with a miR-146a antagomir negative control.

Mice were injected intraperitoneally with D-gal (100 mg/kg/day), saline, or metformin (100 mg/kg/day) for 3 months according to a previously described protocol.^26,50^ Upregulation or downregulation of miR-146a expression in C57 mice was achieved by tail-vein injection of synthetic mmu-miR-146a-5p agomir (250 nmol/kg body weight) or mmu-miR-146a-5p antagomir (250 nmol/kg body weight), with miR-NC used as a negative control (50 nmol/kg body weight). Tail-vein injection was started 1 month after the D-gal and metformin injections. Behavioral tests were conducted at 15 weeks after D-gal injection, and mice were sacrificed at 17 weeks after D-gal injection. The lung and liver tissues were collected for qPCR, immunoblotting, assessment of NAD^+^ levels, and SIRT1 activity assays.

### Statistical analysis

Data from at least three independent experiments are presented as the mean ± SD. Student’s *t* test was used to analyze differences between two groups, and differences between multiple groups were assessed by one-way analysis of variance followed by the Bonferroni post hoc test. *p* < 0.05 was considered statistically significant.

## Supplementary information

SUPPLEMENTAL MATERIAL

## Data Availability

The small-RNA sequencing data included in this study are available in the NCBI Sequence Read Archive (SRA). The SRA under the following accession numbers: SRR10828466, SRR10828465, SRR10828464, SRR10828463, SRR10828462, SRR10828461, SRR10828460, SRR10828459, and SRR10828458.
