# Supplementary material for: miR-146a impedes the anti-aging effect of AMPK via NAMPT suppression and NAD+/SIRT inactivation
Source: Signal Transduct Target Ther. 2022 Mar 4;7:66. doi: 10.1038/s41392-022-00886-3 (PMC8894495; doi:10.1038/s41392-022-00886-3)
Supplement: Supplementary file 1 — SUPPLEMENTAL MATERIAL [file 41392_2022_886_MOESM1_ESM.pdf]

Supplementary Materials

miR-146a impedes the anti-aging effect of AMPK via NAMPT suppression and NAD^{+}/SIRT inactivation

Hui Gong[1], Honghan Chen[1], Peng Xiao[1], Ning Huang[1], Xiaojuan Han[1], Jian Zhang[1], Yu Yang[1], Tiepeng Li[1], Tingting Zhao[1], Haoran Tai[1], Weitong Xu[1], Gongchang Zhang[1], Chuhui Gong[1], Ming Yang[1], Xiaoqiang Tang[2, *], and Hengyi Xiao[1, *]

[1] The Lab of Aging Research, National Clinical Research Center for Geriatrics, State Key Laboratory of Biotherapy, West China Hospital, Sichuan University, 1 Keyuan 4 Road, Gaopeng Avenue, Chengdu, China.
[2] Key Laboratory of Birth Defects and Related Diseases of Women and Children of MOE, State Key Laboratory of Biotherapy, West China Second University Hospital, Sichuan University, Chengdu, China.

***Corresponding Authors**
Hengyi Xiao,
Lab for Aging Research, State Key Laboratory of Biotherapy
West China Hospital, Sichuan University, China.
Tel: +86 28 8516 4023; E-mail: hengyix@scu.edu.cn

Xiaoqiang Tang,
State Key Laboratory of Biotherapy,
West China Second University Hospital,
Sichuan University, Chengdu, China.
E-mail: tangxiaoqiang@scu.edu.cn

This PDF file includes:

Figures. S1 to S10
Tables S1 to S2

Supplementary Table 1. Primers for real-time qRT-PCR

Gene	Forward primer	Reverse primer
(m) *Nampt*	5’-CCTGGTATCCAATTACAGTGGC-3’	5’-CCAAATGAGCAGATGCCCCTAT-3’
(h) *NAMPT*	5’-ATCCTGTTCCAGGCTATTCTGT-3’	5’-CCCCATATTTTCTCACACGCAT-3’
18S	5’-TTGACGGAAGGGCACCACCAG-3’	5’-GCACCACCACCCACGGAATCG-3’
Pri-miR-146a	5’-AATCCAAAAGCACGGCCTCT-3’	5’-TTCGCTGGGATTATGGGGTG-3’
U6	5’TCGCTTCGGCAGCACATATAC-3’	5’-GCGTGTCATCCTTGCGCAG-3’

Supplementary Table 2. Primers for ChIP-qPCR

Gene	Forward primer	Reverse primer
(m) pri-miR-146a promoter	5’- ACGGAGTAAGCTCCAGCCTCCC -3’	5’- ACGGAGTAAGCTCCAGCCTCCC -3’
(m)GAPDH promoter	5’- TACTAGCGGTTTTACGGGCG -3’	5’- TCGAACAGGAGGAGCAGAGAGCGA -3’

Supplementary Figures

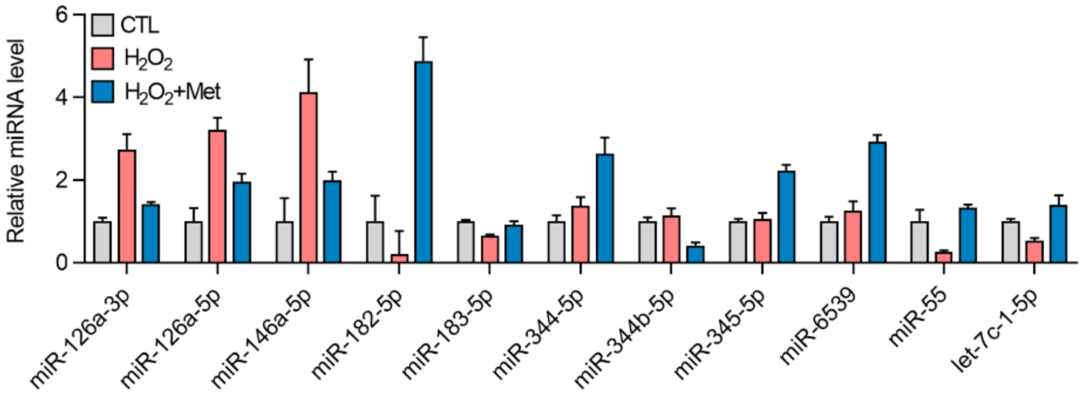

Supplementary Fig.1. Preliminary verification of miRNA expression in senescent and metformin-treated cells. NIH3T3 cells were treated with H_2O_2 (400 μM) for 1h and incubated in complete medium with or without metformin (10 mM) for 3 days, and the miRNA expression in NIH3T3 cells was measured by qRT-PCR (*n*=3).

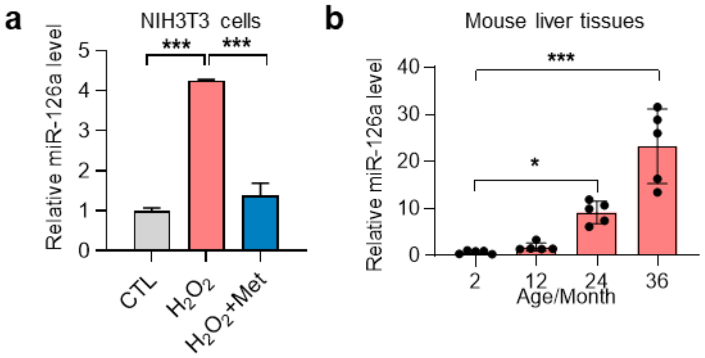

Supplementary Fig.2. miR-126a-5p is increased in senescent cells and aged mice. (a) NIH3T3 cells were treated with H_2O_2 (400 μM) for 1h and incubated in complete medium with or without metformin (10 mM) for 3 days, and miR-126a expression in NIH3T3 cells was measured by qRT-PCR (n=3). **(b)** miR-126a expression in liver from indicated months of mice, analyzed by qRT-PCR (n=5). *p< 0.05, and ***p<0.001.

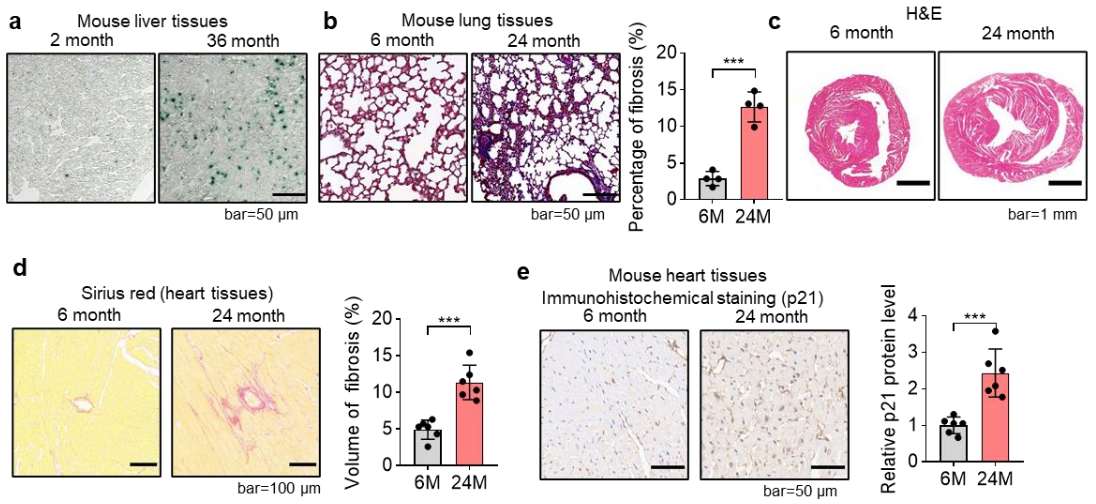

Supplementary Fig.3. Aged mice are accompanied by lung fibrosis and cardiac hypertrophy. (a, b) Representative images of SA-β-gal or Masson staining in liver and lung tissues from indicated months of mice (*n*=4). **(c, d)** Representative images of H&E and Sirius red staining in heart tissues from indicated months of mice (*n*=6). **(e)** Immunohistochemical staining of p21 in heart tissues of aged mice (*n*=6). ***p< 0.001.

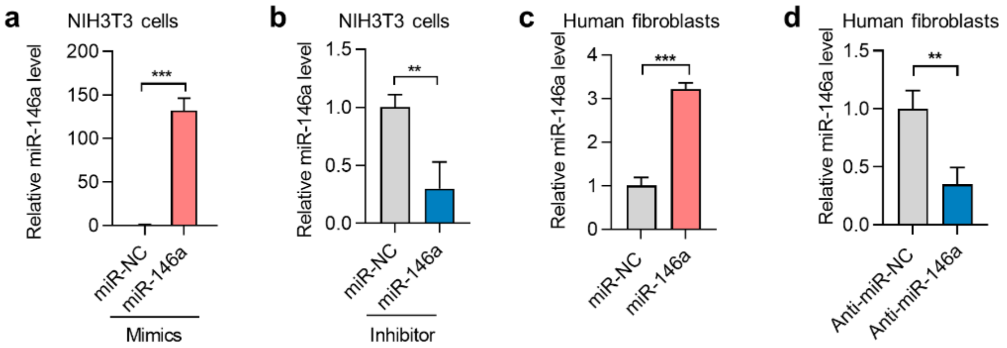

Supplementary Fig.4. miR-146a overexpression or silence in fibroblasts. (a, b) NIH3T3 cells were transfected with miR-146a mimics, miR-146a inhibitor, or respective controls for 24h. miR-146a expression was measured by qRT-PCR (n=3). **(c, d)** Human primary sphenoid sinus mucosa fibroblasts were infected with lentivirus expressing miR-146a, anti-miR-146a, or respective controls. miR-146a expression was measured by qRT-PCR (*n*=3). ***p*< 0.01, and ****p*<0.001.

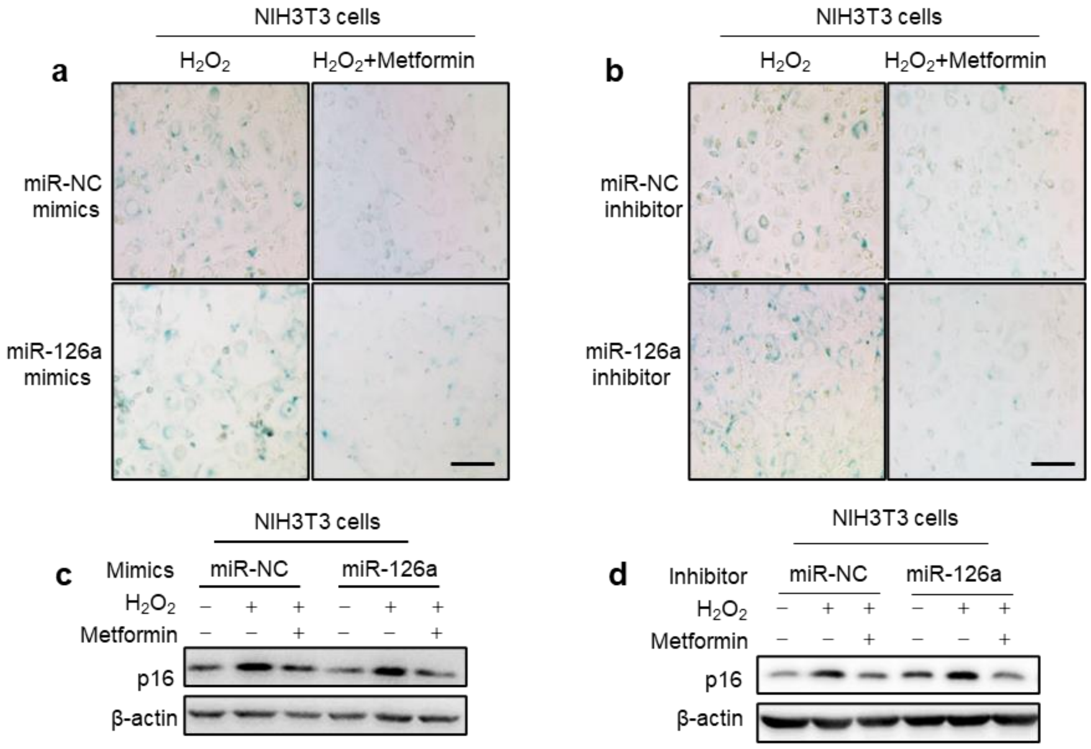

Supplementary Fig.5. miR-126a does not impact the effect of metformin on the alleviation of senescence in cells. NIH3T3 cells were transfected with miR-126a mimics or miR-126a inhibitor for 24h, using miR-NC as the control, then treated with H_2O_2 (400 μM) for 1h and incubated in a complete medium in the presence or absence of metformin (10 mM) for 3 days. **(a, b)** Representative images of SA-β-gal staining, scale bars represent 200 μm. **(c, d)** Representative images of immunoblots for p16. All experiments were repeated three times.

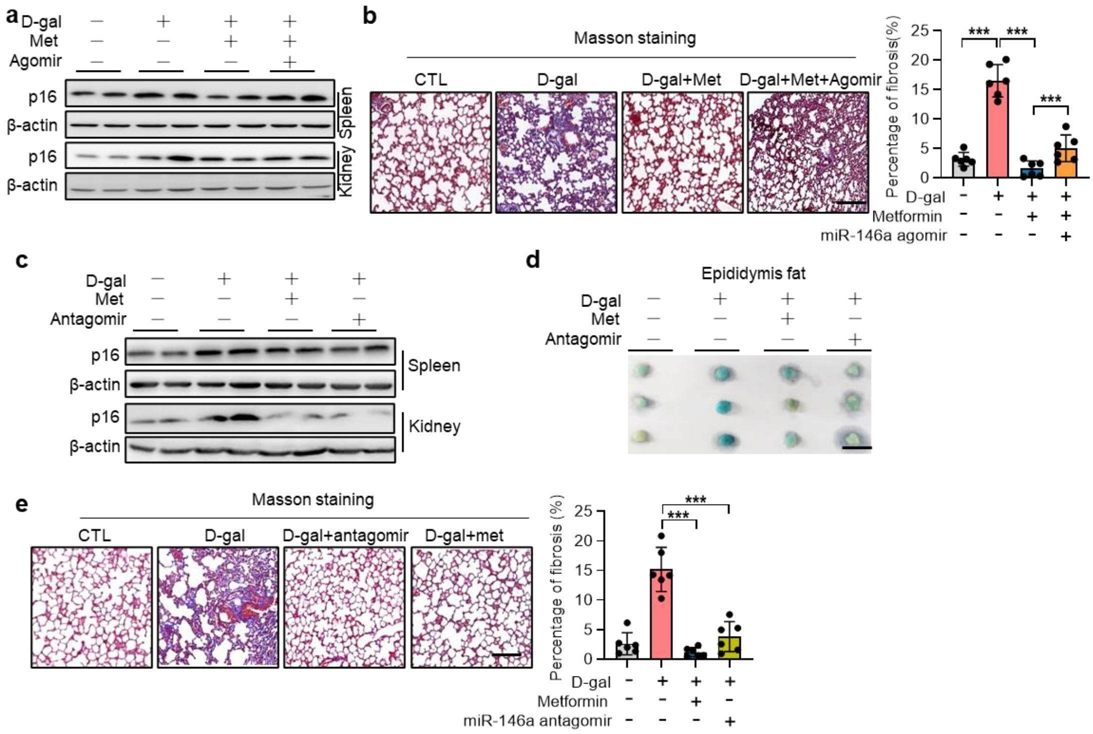

Supplementary Fig.6. miR-146a impedes the protective effect of metformin on aging and related diseases in aged mice. (a-d) The effect of miR-146a agomir. **(a)** Immunoblot images of p16 in spleen and kidney. **(b)** Representative images and quantification of Masson staining in lung tissues (n=6). Scale bar represents 50 µm. **(c-e)** The effect miR-146a antagomir. **(c)** Immunoblot images of p16 in spleen and kidney. **(d)** Representative images of SA-β-gal-staining of Epididymis fat, n=4-6, Scale bars, represent 5 mm. **(e)** Representative images and quantification of Masson staining in lung tissues (n=6). Scale bar represents 50 µm. ***p< 0.001.

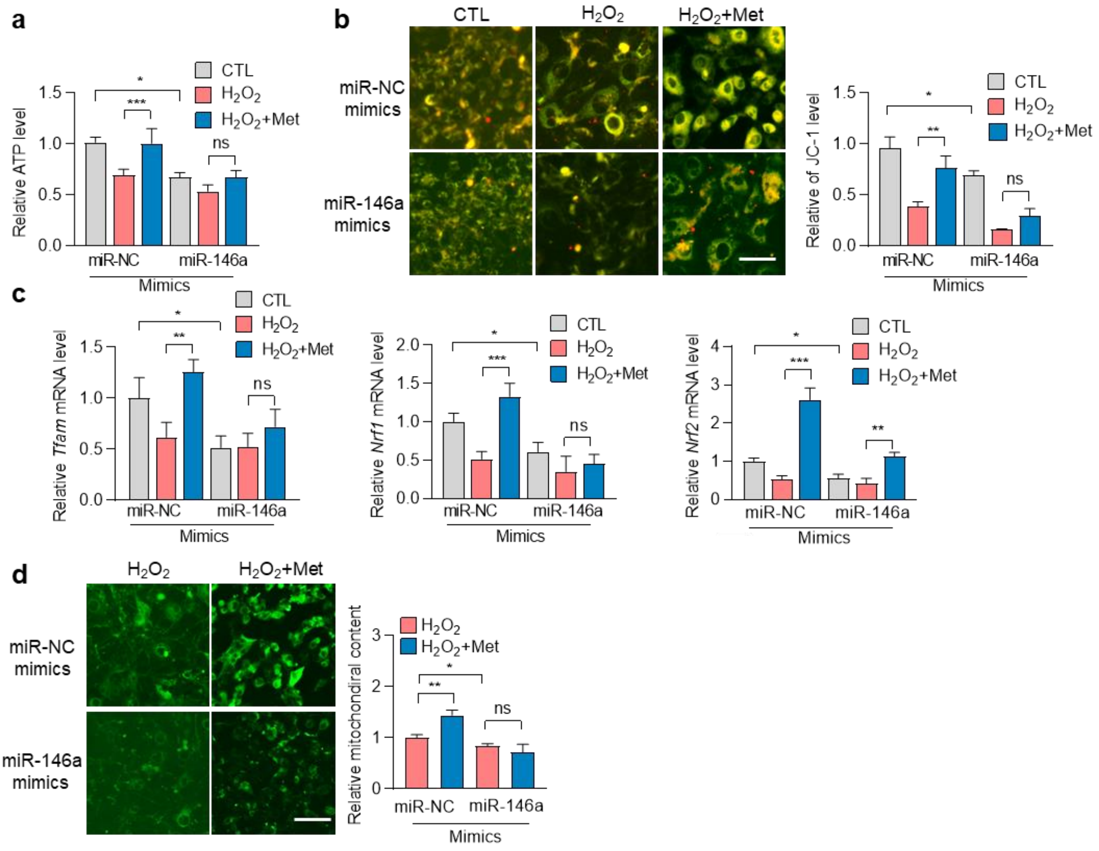

Supplementary Fig.7. miR-146a reduces the effect of metformin on mitochondrial function. NIH3T3 cells were transfected with miR-146a mimics for 24h, using miR-NC as the control, followed by treatment with H_2O_2 and incubation in complete medium for 3 days. **(a)** Intracellular ATP levels were measured (*n*=3). **(b)** Images of JC-1 fluorescence are shown and relative JC-1 level is quantified by a fluorescence microplate reader (n=3). **(c, d)** *Tfam*, *Nrf1*, and *Nrf2* expression were measured by qRT-PCR (*n*=3). **(e)** Mitochondrial content was assessed by MitoTracker Green probe staining. Images of MitoTracker Green are shown, and relative mitochondrial content is quantified by a fluorescence microplate reader (*n*=3). *p<0.05, **p< 0.01, and ***p< 0.001. ns, no significance (p>0.05).

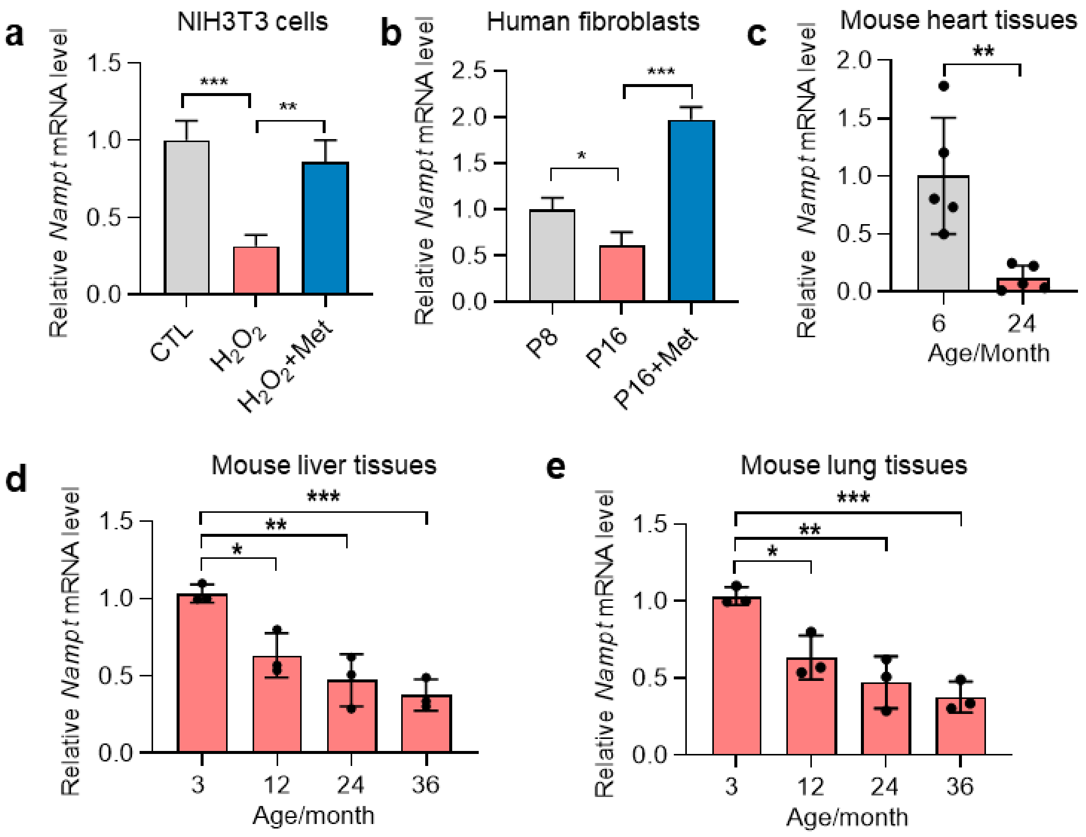

Supplementary Fig.8. NAMPT expression is decreased in senescent cells and aging mice. (a) NIH3T3 cells were treated with H_2O_2 (400 μM) for 1 h and incubated in a complete medium with or without metformin (Met, 10 mM) for 3 days. *Nampt* expression was analyzed by qRT-PCR (n=5). **(b)** Human primary sphenoid sinus mucosa fibroblast induced replicative senescence by passage. *Nampt* expression was analyzed by qRT-PCR (n=3). **(c-e)** *Nampt* expression in the heart (n=5), liver (n = 3), and lung (n=3) tissues from indicated months of mice analyzed by qRT-PCR. *p<0.05, **p<0.01, ***p<0. 001.

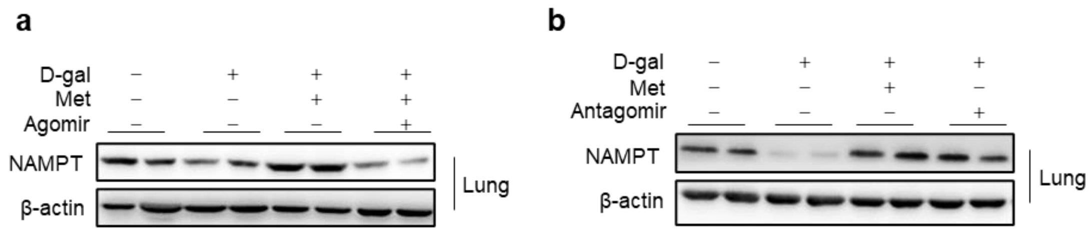

Supplementary Fig.9. miR-146a reduces the effect of metformin on NAMPT expression in lung tissues. (a, b) miR-146a agomir inhibited the up-regulative effect of metformin on NAMPT expression, whereas the miR-146a antagomir showed comparable effects to those of metformin on D-gal-induced aged mice (*n*=6).

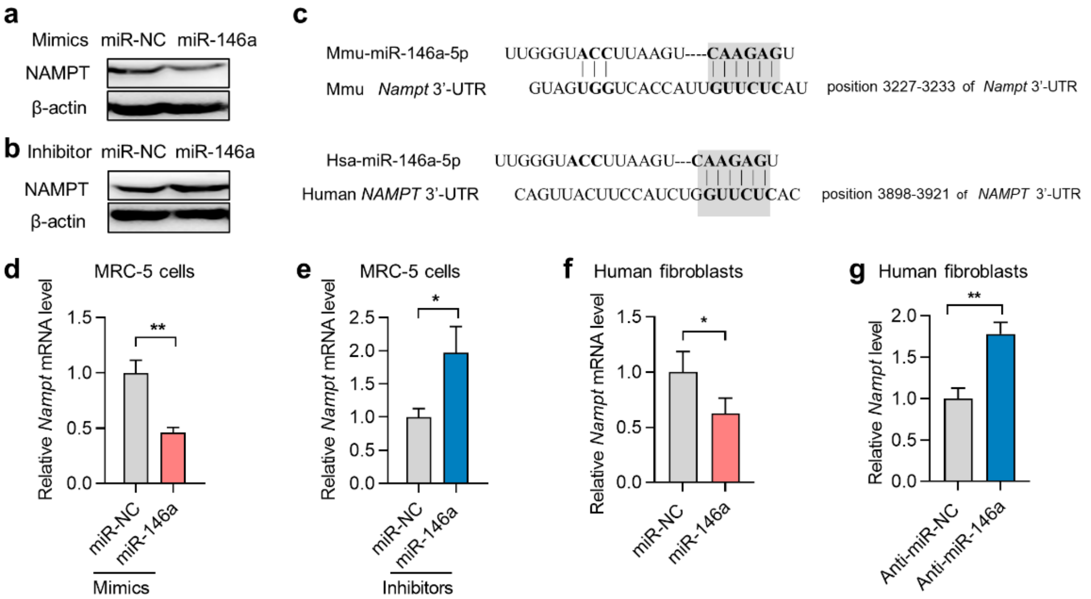

Supplementary Fig. 10. hsa-miR-146a decreases the expression of NAMPT in human cells. **(a-b)** The NAMPT protein level in MRC-5 cells transfected with miR-146a mimics (a) or inhibitors (b). **(c)** The *mmu/hsa* miR-146a sequence, the predicted miR-146a targeting site located in the 3'-UTR of the *Nampt* gene of mice and humans. **(d, e)** The endogenous *Nampt* mRNA level in MRC-5 cells transfected with miR-146a mimics or inhibitors (n=4). **(f, g)** Human primary sphenoid sinus mucosa fibroblast was infected with miR-146a or anti-miR-146a lentivirus, using miR-NC as the control, and *NAMPT* mRNA was measured by qRT-PCR (n=4). *p< 0.05 and **p< 0.01.
